# Supplementary figures and images for: Cyclophosphamide Increases Lactobacillus in the Intestinal Microbiota in Chickens
Source: mSystems. 2020 Aug 18;5(4):e00080-20. doi: 10.1128/mSystems.00080-20 (PMC7438020; doi:10.1128/mSystems.00080-20)

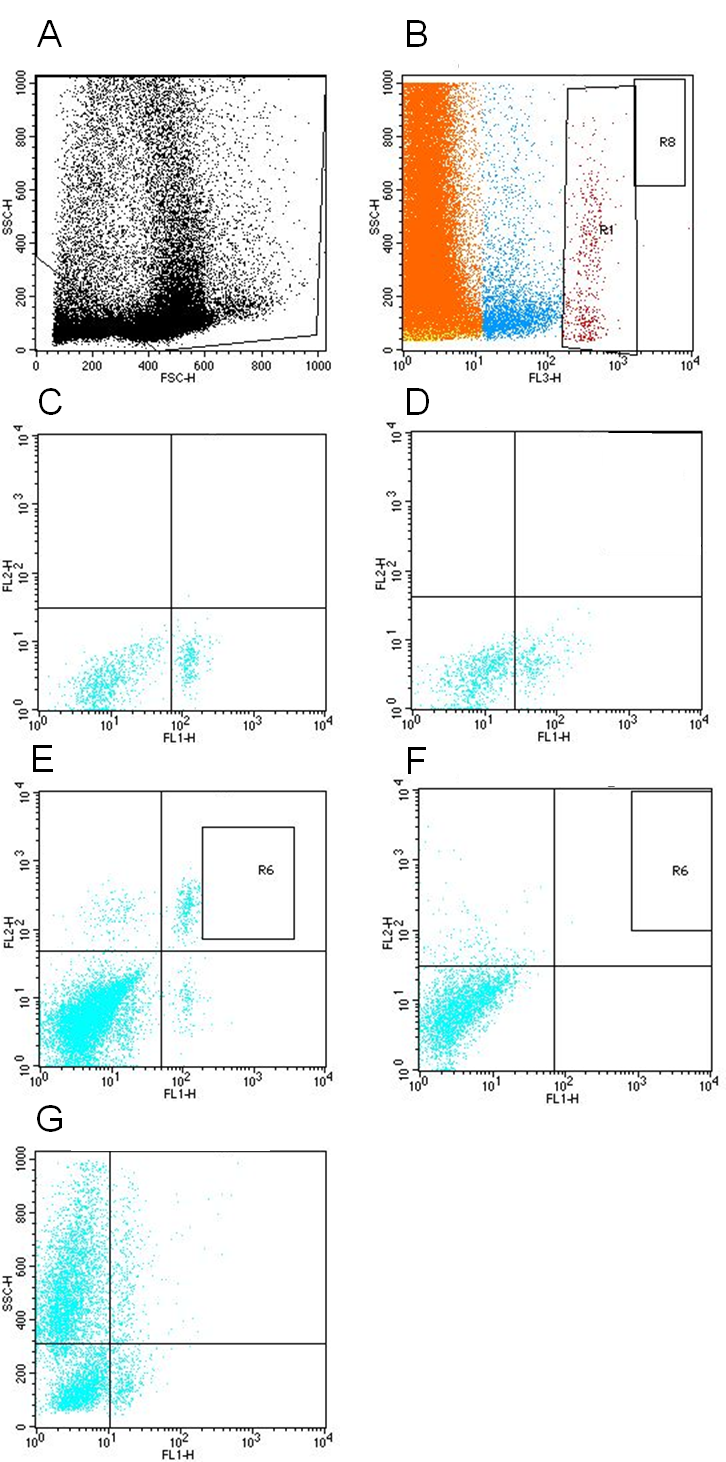

Supplement: FIG S1 [file mSystems.00080-20-sf001.docx]

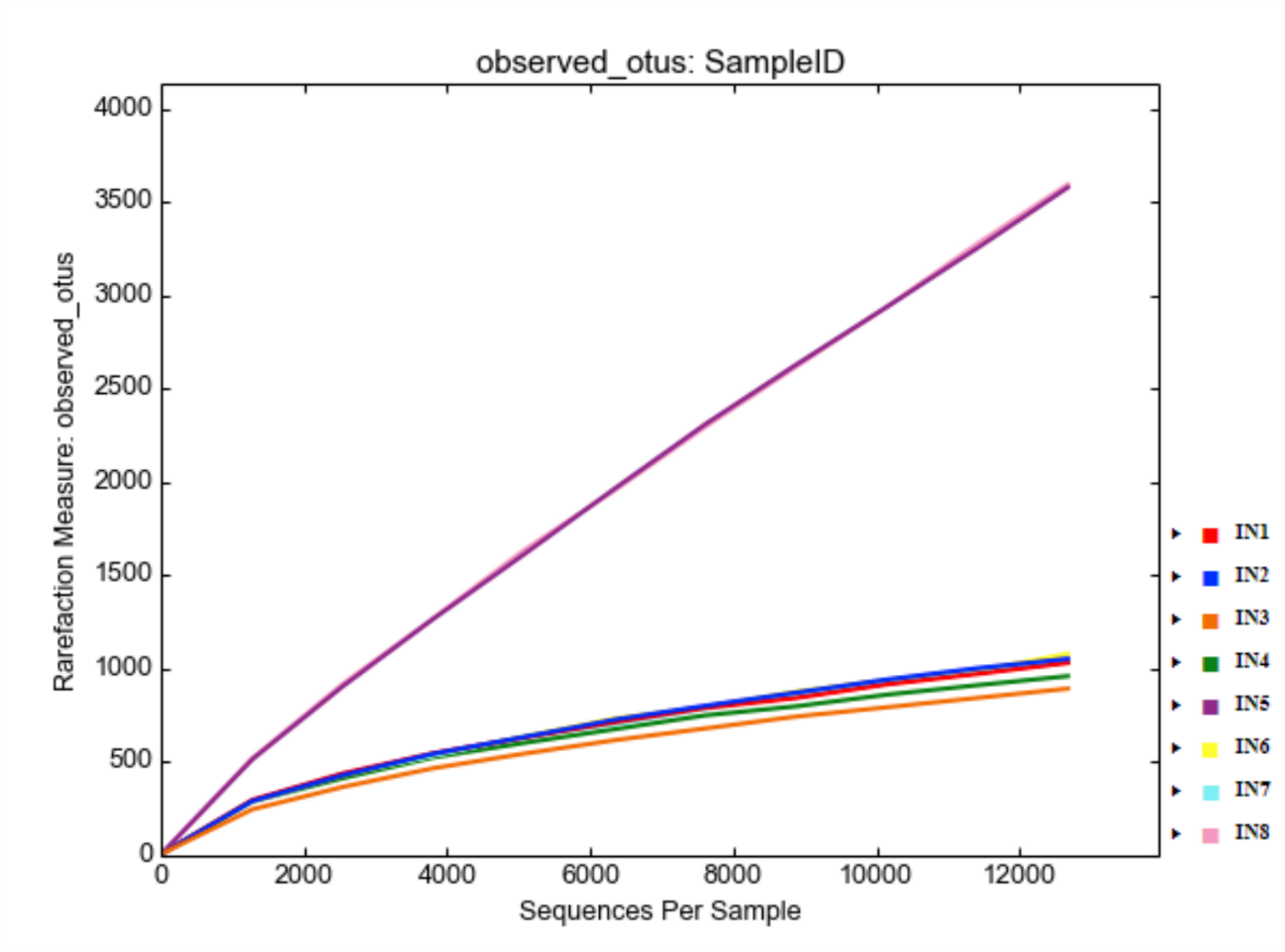

Supplement: FIG S2 [file mSystems.00080-20-sf002.docx]

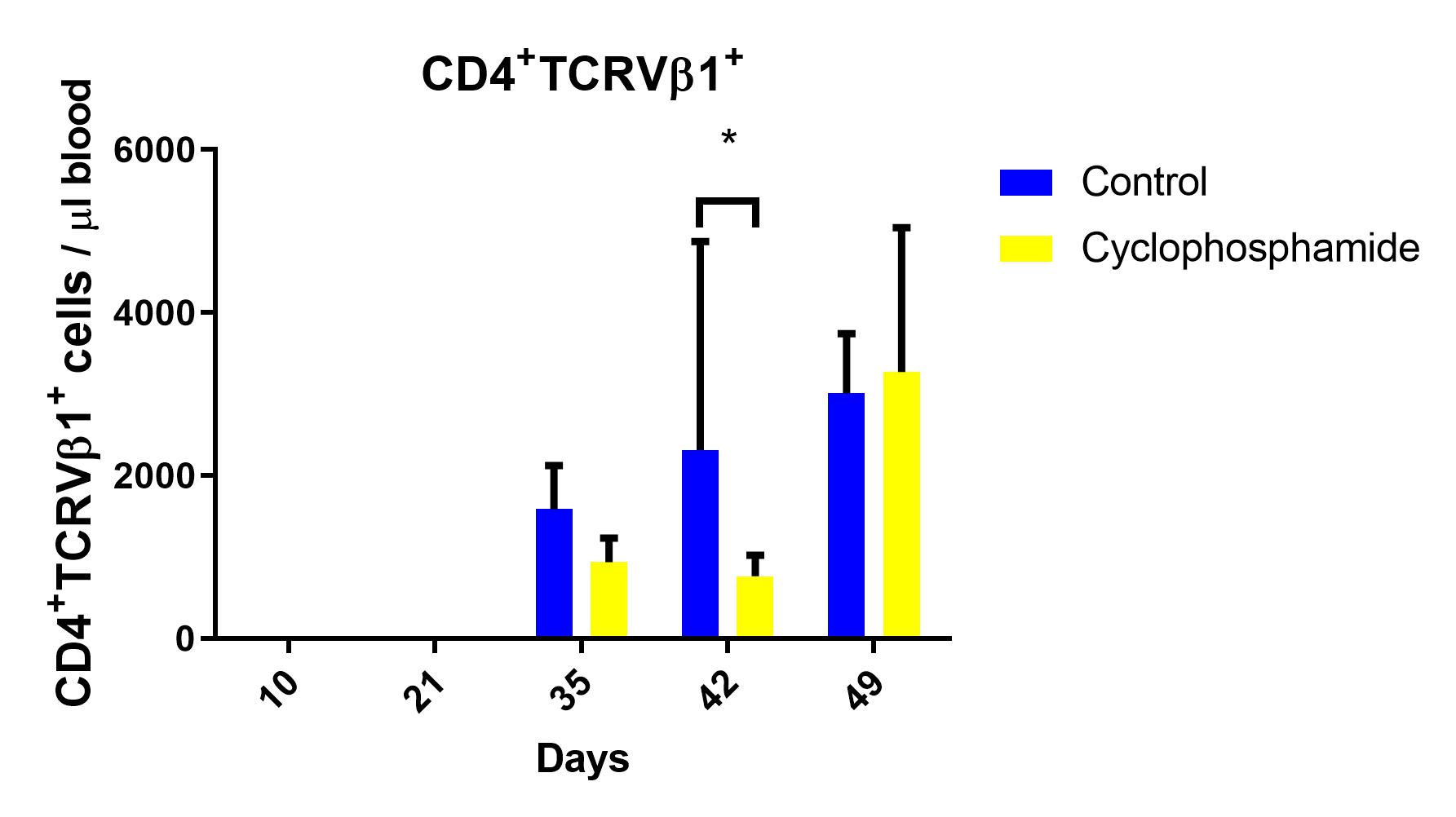

Supplement: FIG S3 [file mSystems.00080-20-sf003.docx]
