# Supplementary material for: Cyclophosphamide Increases Lactobacillus in the Intestinal Microbiota in Chickens
Source: mSystems. 2020 Aug 18;5(4):e00080-20. doi: 10.1128/mSystems.00080-20 (PMC7438020; doi:10.1128/mSystems.00080-20)
Supplement: TABLE S1 [file mSystems.00080-20-st001.docx]

| **Sample detail before normalization** | **Reads** |
| --- | --- |
| IN1: Control | 26520 |
| IN2: Control | 95580 |
| IN3: Control | 12671 |
| IN4: Control | 14253 |
| IN5: Cyclophosphamide | 33456 |
| IN6: Cyclophosphamide | 69050 |
| IN7: Cyclophosphamide | 32666 |
| IN8: Cyclophosphamide | 33424 |
| **Sample summary after normalization** |  |
| Number of Samples | 8 |
| Number of OTUs | 7559 |
| Number of Reads | 101368 |
| Minimum Reads per Sample | 12671 |
| Maximum Reads per Sample | 12671 |
