# Supplementary material for: Cyclophosphamide Increases Lactobacillus in the Intestinal Microbiota in Chickens
Source: mSystems. 2020 Aug 18;5(4):e00080-20. doi: 10.1128/mSystems.00080-20 (PMC7438020; doi:10.1128/mSystems.00080-20)
Supplement: TABLE S2 [file mSystems.00080-20-st002.docx]

| **Phylum** | **Control** | **Cyclophosphamide** |
| --- | --- | --- |
| Firmicutes | 96,02 | 91,43 |
| Bacteroidetes | 0,03 | 2,87 |
| Verrucomicrobia | 0,71 | 2,35 |
| Tenericutes | 1,59 | 1,05 |
| Actinobacteria | 1,04 | 1,25 |
| Proteobacteria | 0,30 | 0,13 |
| Spirochaetae | 0,14 | 0,33 |
| Acidobacteria | 0,14 | 0,42 |
| Gemmatimonadetes | 0,02 | 0,04 |
| Chloroflexi | 0,01 | 0,04 |
| Nitrospirae | 0,01 | 0,03 |
| Cyanobacteria | 0,00 | 0,02 |
| Deferribacteres | 0,00 | 0,02 |
| Planctomycetes | 0,00 | 0,01 |
| Latescibacteria | 0,01 | 0,01 |
