# Supplementary material for: Cyclophosphamide Increases Lactobacillus in the Intestinal Microbiota in Chickens
Source: mSystems. 2020 Aug 18;5(4):e00080-20. doi: 10.1128/mSystems.00080-20 (PMC7438020; doi:10.1128/mSystems.00080-20)
Supplement: TEXT S1 [file mSystems.00080-20-s0001.docx]

Example Pearson correlation

IgA Subdoligranulum Blautia Lachnoclostridium Alistipes Lactobacillus E.coli-Shigella

G1 0.072 44.00 12.50 4.10 8.80 1.30 5.20

G1 0.072 21.20 13.80 10.30 8.90 6.60 5.20

G1 0.071 40.80 15.00 5.40 4.70 2.60 4.20

G1 0.076 17.20 11.00 9.60 4.00 2.80 3.90

# Name of data: grup1

>grup1[,-1]

>grup2<-grup1[,-1]

# New name of data: grup2

>cor(grup2) Distance matrix is calculate.

# New name of data: cor(grup2)

>corre<-cor(grup2)

# Plot: Active the package psych

>corPlot(cor(grup2), diag = TRUE, stars = TRUE, numbers = TRUE, upper = FALSE, las = 2)

Example NMDS

You need two data tables: one with abiotic variables and the second with biotic (microbiota) variables.

LB Mac Mon LT IgA

1 2852 853 3578 856 398

2 1930 731 4963 958 223

3 2563 962 3251 838 324

4 1835 852 4461 773 235

5 560 389 1215 286 126

6 662 462 1432 212 96

7 873 387 968 186 84

8 418 423 895 196 97

>read.table("testA.txt", header = T)->abiotic

>read.table("testB.txt", header = T)->biotic

>decostand(abiotic, method = "standardize")->abiotics

>ord <- metaMDS(biotic)

>fit <- envfit(ord, abiotics, perm = 999)

>plot(ord)

>plot(fit)

>plot(fit, p.max = 0.05, col = "red")

>treat=c(rep("Treatment1",4),rep("Treatment2",4))

>plot(ord, type = "n", main ="Non-metric multidimensional scaling plot")

>ordiellipse(ord,groups=treat,kind="ehull",draw="polygon",col="grey90",label=F)

>orditorp(ord,display="species",col="red",air=0.01, cex = 0.7)

>orditorp(ord,display="sites",col=c(rep("dark green",4),rep("dark magenta",5)),air=0.01,cex=1.25)

>plot(fit)
