# Supplementary material for: Cyclophosphamide Increases Lactobacillus in the Intestinal Microbiota in Chickens
Source: mSystems. 2020 Aug 18;5(4):e00080-20. doi: 10.1128/mSystems.00080-20 (PMC7438020; doi:10.1128/mSystems.00080-20)
Supplement: TEXT S2 [file mSystems.00080-20-s0002.docx]

**Flow cytometry gating strategy**

Blood samples were divided to allow concomitant staining with antibodies (or phagocytosis beads) with the same fluorophore. Following antibody staining of cells, samples were gated on the flow cytometer based on size (FSC) and granularity (SSC) (Figure S1 A).

All samples were stained for CD45 (pan-leucocyte marker, clone LT-40), which was SPRD-coupled. CD^45bright+^ cells were gated and then analysed for expression of other cell markers (Figure S1 B). Small debris were excluded from analysis. Leukocyte subsets were then quantified in the specific fluorophore channel for the given antibody. Anti-CD4 (clone CT-4) and anti-CD8 alpha (CT-8) were FITC-coupled; anti-TCR Vβ1 (TCR2), and anti-Bu-1 (Av20) were PE-labelled. At least 10,000 CD^45bright+^ cells were counted. For identification of phagocytic cells, gating of leukocytes was similar to that of antibody-stained cells (Figure S1 A and B). However, phagocytic cells were quantified based on the fluorescence of internalized beads. Cells were also discriminated according to SSC to differentiate granulocytes from monocytic blood cells (Figure S1 D). Single-colour controls were used for eliminating spectral overlaps. All fluorophores were excited with an argon laser.
